# Retraction Note: Cardioprotective activity of placental growth factor in a rat model of acute myocardial infarction: nanoparticle-based delivery versus direct myocardial injection

**DOI:** 10.1186/s12872-015-0019-z

**Published:** 2015-03-26

**Authors:** Zhe-xin Lu, Li-li Mao, Feng Lian, Jun He, Wen-tian Zhang, Chen-yang Dai, Song Xue, Wei-gen Lu, Hong-sheng Zhu

**Affiliations:** Department of Cardiovascular Surgery, Ren Ji Hospital, School of Medicine, Shanghai Jiao Tong University, 160 Pu-Jian Rd, Shanghai, 200127 People’s Republic of China

## Retraction

The Publisher and Editor regretfully retract this article [1] because the peer-review process was inappropriately influenced and compromised. As a result, the scientific integrity of the article cannot be guaranteed. A systematic and detailed investigation suggests that a third party was involved in supplying fabricated details of potential peer reviewers for a large number of manuscripts submitted to different journals. In accordance with recommendations from COPE we have retracted all affected published articles, including this one. It was not possible to determine beyond doubt that the authors of this particular article were aware of any third party attempts to manipulate peer review of their manuscript.

## References

[1] Lu ZX, Mao LL, Lian F, He J, Zhang WT, Dai CY (2014). BMC Cardiovasc Disord.

